# Assessment of Lifestyle and Dietary Patterns Among Students at the University of Human Development in Sulaimani City: A Cross‐Sectional Study 2025–2026

**DOI:** 10.1002/hsr2.72125

**Published:** 2026-03-19

**Authors:** Cheeman Salih Kakabra, Maisam Hama Murad Majeed, Ari Jamal Hassan, Abdulmalik Fareeq Saber, Sara Noori Mohammad, Bushra Jabbar Hamarashid, Bayan Omar Sharif, Prusha Soran Karim, Ara Othman Muhammed, Shan Hamza Muhammad, Savyar Ghafur Rashid, Bashir Mohammed Rasul

**Affiliations:** ^1^ Department of Community Health Nursing, College of Nursing University of Sulaimani Sulaimani Kurdistan Region Iraq; ^2^ Department of Adult Nursing, College of Nursing University of Sulaimani Kurdistan Region Iraq; ^3^ Department of Psychiatric Nursing, School of Nursing & Midwifery Tehran University of Medical Sciences Tehran Iran; ^4^ College of Nursing Lebanese French University Erbil Kurdistan Region Iraq; ^5^ Department of Statistics and Informatics, College of Administration and Economics University of Sulaimani Sulaimani Kurdistan Region Iraq; ^6^ College of Nursing University of Human Development Sulaimani Kurdistan Region Iraq; ^7^ Department of Adult Nursing, College of Nursing University of Raparin Rania Kurdistan Region Iraq

**Keywords:** behavior, dietary patterns, health promotion, university students

## Abstract

**Background and aim:**

In Sulaimani, Iraq, university students often engage in lifestyle behaviors that may influence their dietary habits, nutritional health, and overall well‐being. This study aimed to assess the lifestyle characteristics, dietary patterns, and associated predictors among students at the University of Human Development.

**Method:**

This cross‐sectional study was conducted from February 11th to February 20th, 2025, across eight academic departments at the University of Human Development, using Convenience sampling technique. The questionnaire included demographic information, lifestyle indicators, sleep and activity patterns, medical history, and a detailed food frequency section. Statistical analysis was performed using SPSS version 26 (IBM Corp., Armonk, NY). Chi‐square tests, ANOVA, and binary logistic regression were conducted to assess associations between dietary patterns and demographic, behavioral, and health‐related variables.

**Results:**

A total of 330 university students participated in the study. Fruit and vegetable consumption was suboptimal, while refined carbohydrate intake was high (white rice: 46.36% daily; white bread: 36.06% daily). Students who always slept after midnight had higher odds of following a non‐routine dietary pattern (OR = 2.44; 95% CI: 1.01–5.89; *p* = 0.047). Those who never achieved 7–8 h of sleep were also more likely to report non‐routine dietary behavior (OR = 2.07; 95% CI: 1.00–4.30; *p* = 0.050). Regular gym or swimming activity was associated with lower odds of non‐routine dietary patterns (OR = 0.51; 95% CI: 0.31–0.85; *p* = 0.01). Additionally, consistent electronic device use at bedtime was associated with increased odds of dietary irregularity (OR = 2.03; 95% CI: 1.01–4.13; *p* = 0.048), and a family history of obesity was associated with higher odds (OR = 1.75; 95% CI: 1.00–3.07; *p* = 0.049).

**Conclusions:**

This cross‐sectional study found that university students in Sulaimani exhibited suboptimal dietary patterns associated with poor sleep hygiene, low physical activity, excessive screen time, and a family history of obesity. Future longitudinal and intervention‐based studies are needed to establish causal relationships and evaluate the effectiveness of campus‐based health promotion programs.

## 1 Introduction

Lifestyle and dietary behaviors among young adults have emerged as a major global public health concern, with far‐reaching implications for the prevention of noncommunicable diseases (NCDs). Worldwide, up to 80% of young adults report engaging in unhealthy eating patterns and sedentary lifestyles, contributing to rising rates of obesity, diabetes, and cardiovascular disease [1, 2]. In the Middle East, rising obesity rates among youth—reported at 32% in Iraq and nearly 40% in some Gulf countries—reflect the growing impact of unhealthy lifestyle behaviors [3]. Although these behaviors are widespread across populations, they are especially pronounced among university students living in urbanized settings where fast food, digital distractions, and academic pressures dominate [4]. Among Iraqi university students, a recent national survey found that over 65% consume fast food more than three times per week, and more than 70% do not meet WHO physical activity recommendations [5]. A global strategy to improve adolescent nutrition and physical activity (notably through school and university policies) was launched by WHO in 2004, yet college‐age populations were not the primary focus [6]. Formal targets aimed at improving youth dietary quality and activity levels were only included later in the 2025 Global Action Plan on Physical Activity and Healthy Diets [7, 8]. Consequently, university students in transitional societies have become emblematic of a global shift toward poor health behaviors [9].

Students typically experience a reduction in structured meals and an increase in snacking, with symptoms such as fatigue, concentration difficulties, and weight fluctuations commonly reported [10]. In a study conducted at Salahaddin University in Erbil, 62% of students reported skipping breakfast regularly, while nearly half consumed energy drinks at least once a week [11]. However, students in health‐related programs tend to adopt slightly healthier choices, highlighting variability across academic disciplines [12]. Those dealing with academic overload, economic stress, or lack of parental oversight may be at greater risk of developing chronic behavioral patterns linked to metabolic syndromes, obesity, and mental health issues [13, 14]. The compounded effect of low physical activity, irregular meals, and high fast‐food intake could, over time, contribute to an increased risk of NCDs and poor academic outcomes in over 70% of this population [15].

First‐line prevention of lifestyle‐related disorders relies on early screening and public health education, which have shown effectiveness in promoting behavior change among young adults, especially within academic institutions [16]. Peer‐led health education initiatives in Jordan and Lebanon demonstrated improvements in diet awareness and fruit intake frequency by 20%–30% after just 8 weeks [17]. Yet most interventions remain short‐term, with inconsistent monitoring, and are rarely integrated into university curricula or policies [18]. While digital tools and app‐based wellness programs offer convenience and broad reach, they are often limited by low engagement rates, inconsistent usage, and cultural mismatches in diverse student bodies [19, 20]. Integrated programs that combine personalized dietary advice with peer‐group physical activity initiatives have been shown to increase student participation and sustainability [21, 22]. Despite these innovations, only 17% of Iraqi universities report having a structured student wellness center, and very few offer nutrition counseling on campus [23]. These strategies also address logistical barriers, such as lack of time or access to healthy food and exercise spaces, which are frequently reported by students [24, 25]. Nonetheless, barriers related to affordability, motivation, and institutional support continue to hinder the widespread adoption of health‐promoting behaviors [26].

Sulaimani City, like many urban centers in Iraq, is witnessing a rapid nutrition transition characterized by increased processed food consumption and physical inactivity among youth [27]. The University of Human Development offers a representative academic environment for assessing such changes, as its students come from diverse socioeconomic and academic backgrounds. Previous small‐scale studies have indicated high rates of sugar‐sweetened beverage consumption and insufficient physical activity among Kurdistan university students [28]. To date, no comprehensive study has simultaneously assessed dietary habits, sleep hygiene, physical activity, screen time, and their interrelationships among students at this institution. Therefore, the objective of the current cross‐sectional study is to evaluate the dietary and lifestyle patterns among students at the University of Human Development in Sulaimani during the academic year 2025–2026, and to identify demographic, behavioral, and health‐related predictors of dietary regularity.

## 2 Research Question

How are the lifestyle and dietary patterns of students at the University of Human Development in Sulaimani City characterized during the academic year 2025–2026?

## 3 Methods

### 3.1 Study Design, Setting, Period, and Sampling

This cross‐sectional study was conducted in Sulaimani, Iraq, from February 11th to February 20th, 2025, using a convenience sampling technique. All eight academic departments at the University of Human Development (UHD) were included to ensure representation across both health‐related and non‐health‐related disciplines: Nursing, Medical Laboratory Sciences (MLS), Information Technology (IT), Computer Science (CS), Law, English Language, Business Administration, and Accounting. Data collection was conducted during scheduled class hours with instructor's cooperation. Participation was highest in Nursing (*n* = 93; 28.18%) and MLS (*n* = 85; 25.76%), followed by IT (*n* = 47; 14.24%), Law (*n* = 45; 13.64%), and smaller contributions from Business Administration, English Language, Accounting, and Computer Science. This variation likely reflects differences in class sizes and willingness to engage in a health‐focused survey.

### 3.2 Sample Size

The sample size was determined using Cochran′s formula for finite populations, with a 95% confidence level, 5% margin of error, and an estimated population proportion of 60%, resulting in a required sample size of 369 students. Of these, 330 participants agreed to participate and completed the questionnaire (response rate: 89.4%), while the remaining individuals either declined or submitted incomplete responses. Although the achieved sample fell slightly below the target, post‐hoc power analysis indicated that a sample of 330 provides adequate statistical power (> 80%) to detect moderate effect sizes (OR ≥ 1.5) in binary logistic regression with the number of predictors used in this study, and the margin of error increased only marginally from 5.0% to approximately 5.3%, which is unlikely to substantially affect the validity of the findings.

### 3.3 Inclusion/Exclusion

The inclusion criteria for participants included undergraduate students of both genders from any academic year and department at UHD. Participants were required to be actively enrolled and present during data collection. On the other hand, students who refused to participate, were exchange students, or had incomplete responses were excluded from the study (*n* = 22).

### 3.4 Study Tools and Data Collection

The questionnaire was divided into nine main parts. The first part collected sociodemographic data including age, gender, academic department, year of study, place of residence, marital status, monthly income, and ethnicity. The remaining sections comprised a structured, self‐administered 45‐item tool organized thematically: Body Mass Index, Past Medical History, Family History, Weight Management Practices, Sleep Hygiene, Physical Activity, Nutritional Regimen, and Dietary Patterns Inventory. The dietary section used a 5‐point Likert scale to assess healthy and unhealthy food frequencies. The 45‐item questionnaire was developed by the research team based on previously validated dietary and lifestyle assessment instruments. It was originally constructed in English and translated to local native language using backward translation. reviewed by three content experts for face and content validity. The complete questionnaire is provided as a supplementary file (Appendix A). For any unclear items, explanations were offered by the researchers to ensure accurate and consistent understanding across participants in accordance with the STROBE (Strengthening the Reporting of Observational Studies in Epidemiology) guidelines for cross‐sectional studies. Each participant was allotted approximately 10–15 min to complete the form.

### 3.5 Pilot Study

The study questionnaire was initially piloted with a group of 30 students enrolled in various UHD departments. The pilot was conducted between January 5th and February 5th, 2025, to assess internal consistency and item clarity. Cronbach′s alpha was calculated as 0.87 [29], indicating very good internal reliability. Feedback from the pilot phase led to minor language revisions, and data from the pilot participants were excluded from the final analysis.

### 3.6 Measures

#### 3.6.1 Sociodemographic Characteristics

The first section of the questionnaire included sociodemographic characteristics such as age, gender, academic department, level of study, place of residence, marital status, family income, and ethnicity.

#### 3.6.2 Lifestyle and Dietary Assessment Tool

To assess lifestyle and dietary patterns among university students, we used a structured 45‐item questionnaire. This included three items on sleep hygiene, one item on physical activity, and two items on dietary planning. For dietary consumption, 20 food items were assessed using a frequency scale. Healthy items (e.g., fruits, vegetables, lean proteins) were scored from 0 (never) to 4 (daily), while unhealthy foods (e.g., sweets, processed snacks) were reverse‐scored. Fruit and vegetable intake received additional weighting. Total nutrition scores were categorized into poor (0–25), moderate (26–50), and optimal (51–80) based on a tertile‐based approach, which is widely used in dietary pattern research when validated cut‐off points are unavailable [30]. While these thresholds were determined by the distribution of scores within the study sample rather than externally validated benchmarks, this approach is consistent with prior studies assessing dietary quality among university populations. The absence of a validated scoring framework specific to this context is acknowledged as a limitation. Medical and family history sections screened for eight conditions and three familial risks, including thyroid disorders, diabetes, and obesity. Weight management practices and BMI were also assessed using WHO classification based on self‐reported height and weight.

### 3.7 Ethical Approval and Inform Consent

This study followed the Institutional Research Ethics Board and the Declaration of Helsinki guidelines. Ethical approval for the study was obtained from the Research Ethics Committee of the College of Nursing at UHD in January 2025. Written informed consent was obtained from all participants prior to questionnaire administration. Administrative permissions were secured from department heads, and confidentiality was maintained through anonymized data handling and secure data storage protocols.

### 3.8 Statistical Analysis

Data were summarized and reported using frequencies and percentages for categorical variables and mean ± standard deviation for continuous variables. Group comparisons between categorical variables were conducted using chi‐square tests. Prior to conducting ANOVA, normality was assessed using the Shapiro–Wilk test and visual inspection of Q‐Q plots, and homogeneity of variances was evaluated using Levene′s test. For logistic regression, multicollinearity among predictors was assessed using variance inflation factors (VIF), all of which were within acceptable limits (VIF < 5). All assumptions were satisfactorily met before proceeding with the analyses. Differences in continuous dietary intake variables across BMI categories were assessed using one‐way analysis of variance (ANOVA), followed by Tukey post‐hoc tests where applicable. Associations between lifestyle behaviors, demographic factors, and the likelihood of following non‐routine dietary programs were analyzed using binary logistic regression models, adjusting for relevant confounders such as age, gender, and income. All tests were two‐sided with an a priori significance level of *α* = 0.05. Given the exploratory nature of this study, no formal correction for multiple comparisons (e.g., Bonferroni) was applied. Instead, emphasis was placed on effect sizes and confidence intervals throughout the manuscript to allow readers to evaluate practical significance beyond p‐values alone. Data analysis was performed using SPSS version 26 (IBM Corp., Armonk, NY), and statistical significance was determined at *p* < 0.05. Statistical reporting in this manuscript follows the recommendations of Assel for the proper analysis, reporting, and interpretation of clinical research statistics [31, 32].

## 4 Results

### 4.1 Demographic Characteristics

A total of 330 students participated in this study assessing lifestyle and dietary patterns at the University of Human Development. The mean age of participants was 20.94 ± 1.82 years, with the largest age group being 18–20 years (43.33%, *n* = 143), closely followed by 21–23 years (43.03%, *n* = 142). Males represented a higher proportion of the sample at 62.73% (*n* = 207) compared to females at 37.27% (*n* = 123). The majority of students were enrolled in Nursing (28.18%, *n* = 93) and Medical Laboratory Science (25.76%, *n* = 85), while smaller groups belonged to departments such as Information Technology (14.24%, *n* = 47) and Law (13.64%, *n* = 45). Regarding academic progression, most students were in their Fourth Year (34.55%, *n* = 114) and Second Year (25.15%, *n* = 83). A significant majority resided inside Sulaimani City (85.45%, *n* = 282) and were single (87.58%, *n* = 289). In terms of socioeconomic status, 45.45% (*n* = 150) reported a monthly family income above $770, while 41.82% (*n* = 138) earned between $385 and $770. For BMI, 64.55% (*n* = 213) had normal weight, whereas 23.94% (*n* = 79) were overweight and 5.76% (*n* = 19) fell into both underweight and obese categories respectively. Detailed demographics are presented in Table 1.

**Table 1:** Socio‐demographic characteristics of the participants (*n* = 330).

No.	Variable	Characteristics *n* = 330	F	%
1.	Age group (years)	18–20	143	43.33
		21–23	142	43.03
		24–26	39	11.82
		27–29	6	1.82
		Mean ± SD	20.94 ± 1.82	
2.	Gender	Male	207	62.73
		Female	123	37.27
3.	Academic Department	Accounting	13	3.94
		Business Administration	20	6.06
		Computer Science	12	3.64
		Information Technology (IT)	47	14.24
		English Language	15	4.55
		Law	45	13.64
		Medical Laboratory Science (MLS)	85	25.76
		Nursing	93	28.18
4.	Academic stage	First Year	76	23.03
		Second year	83	25.15
		Third year	57	17.27
		Fourth year	114	34.55
5.	Place of residence	Inside Sulaimani City	282	85.45
		Outside Sulaimani city	48	14.55
6.	Marital status	Single	289	87.58
		Married	32	9.70
		Divorced	9	2.73
7.	Monthly family income (USD)	Less than $385	42	12.73
		$385–770	138	41.82
		More than $770	150	45.45
8.	Body mass index (BMI, kg/m²)	Underweight (< 18.5)	19	5.76
		Normal weight (18.5–24.9)	213	64.55
		Overweight (25–29.9)	79	23.94
		Obese (30–34.9)	19	5.76

Abbreviations: BMI = Body Mass Index (calculated as weight in kilograms divided by height in meters squared, kg/m²), F = Frequency, IT = Information Technology, MLS = Medical Laboratory Science, % = Percentage.

### 4.2 Medical History and Weight Management Behaviors

The results revealed that the majority of participants did not report having any chronic medical conditions. Type 2 Diabetes Mellitus was present in only 2.67% (*n* = 8) of students, while 97.33% (*n* = 292) had no such history. Similarly, Hypothyroidism was reported by 7.67% (*n* = 23), and Hyperthyroidism by 3.00% (*n* = 9). Among female‐related conditions, 16.33% (*n* = 49) reported having polycystic ovarian syndrome (PCOS). H. pylori infection was noted in 17.00% (*n* = 51) of participants, making it the most common gastrointestinal issue, followed by gastric ulcer at 5.33% (*n* = 16) and celiac disease at 4.67% (*n* = 14). Regarding weight management, the use of medications for weight gain and weight loss was rare, reported by only 3.03% (*n* = 10) and 3.33% (*n* = 11) respectively. Only 1.21% (*n* = 4) of participants had undergone weight loss surgery, indicating that surgical intervention was extremely uncommon in this population. For further details, see Table 2.

**Table 2:** Past medical history and weight management practices of the participants.

Domain	Variable	Response	Frequency (F)	Percentage (%)
Past medical history	Type 2 diabetes mellitus	Yes	8	2.67
		No	292	97.33
	Hypothyroidism	Yes	23	7.67
		No	277	92.33
	Hyperthyroidism	Yes	9	3.00
		No	291	97.00
	Polycystic ovarian syndrome (PCOS)	Yes	49	16.33
		No	251	83.67
	Asthma	Yes	15	5.00
		No	285	95.00
	*Helicobacter pylori* (H. pylori) Infection	Yes	51	17.00
		No	249	83.00
	Gastric ulcer	Yes	16	5.33
		No	284	94.67
	Celiac disease	Yes	14	4.67
		No	286	95.33
Weight management	Use of medication for weight gain	Yes	10	3.03
		No	320	96.97
	Use of medication for weight loss	Yes	11	3.33
		No	319	96.67
	Underwent surgery for weight loss	Yes	4	1.21
		No	326	98.79

Abbreviations: BMI = Body Mass Index; H. pylori = Helicobacter pylori; PCOS = Polycystic Ovarian Syndrome. Past Medical History percentages are calculated based on n = 300 (30 incomplete responses); Weight Management percentages are based on n = 330.

### 4.3 Family History, Sleep, Physical Activity, and Diet

The results revealed that one‐third of students (34.55%) had a family history of type 2 diabetes, while 20.30% reported thyroid‐related conditions among family members. Regarding sleep habits, a large majority (over 94%) reported going to bed after midnight, either always or sometimes, and only 27.27% consistently slept 7–8 h daily. Electronic device use at bedtime was also high, with nearly half (49.39%) always engaging in this behavior. Physical activity levels were suboptimal, as 35.76% reported no regular exercise, though others engaged in walking or gym/swimming. Dietary patterns showed that half of the students (50.00%) consumed more than three meals per day, while only a small portion (3.03%) followed a diet supervised by a nutritionist. For more details, refer to Table 3.

**Table 3:** Health behavior and family history characteristics of the Participants (*n* = 330).

Domain	Variable	Category/Response	Frequency (F)	Percentage (%)
Family medical history	Family history of type 2 diabetes mellitus	—	114	34.55
	Family history of thyroid disorder	—	67	20.30
	Family history of obesity	—	48	14.55
	No family medical history	—	101	30.61
Sleep patterns	Sleep after 12:00 AM	Always	138	41.82
		Sometimes	174	52.73
		Never	18	5.45
	Sleep 7–8 h per day	Always	90	27.27
		Sometimes	214	64.85
		Never	26	7.88
	Use of electronic devices at bedtime	Always	163	49.39
		Sometimes	137	41.52
		Never	30	9.09
Physical activity	Regular physical activity ( ≥ 30 min, 5 days/week)	None	118	35.76
		Walking	103	31.21
		Gym or Swimming	109	33.03
Dietary patterns	Meals per day	One Meal	19	5.76
		Two Meals	146	44.24
		More Than Three Meals	165	50.00
	Diet program type	Under Nutritionist Schedule	10	3.03
		Self‐Designed Program	81	24.55
		Normal Eating Pattern	239	72.42

*Note:* Sleep patterns and dietary behaviors are categorized based on frequency. Physical activity is based on the self‐reported practice of ≥ 30 min/day for 5 days/week. Family income data are presented in USD elsewhere.

### 4.4 Food Consumption Patterns

The results showed that chicken was the most frequently consumed protein source, with 47.58% of students eating it three times a week or more. In contrast, lean meat and fish had much lower intake, with 56.97% and 79.39%, respectively, consuming them once a week or less. Consumption of boiled eggs was generally low, with 42.12% not eating them at all. Among plant‐based foods, legumes and dairy products had moderate intake, with around one‐third of students consuming them at least 3 times per week. Notably, fruits had relatively good intake, with 46.36% eating them three times a week or more, while vegetable consumption was less frequent, with only 32.73% consuming them at that rate. For more details, refer to Table 4.

**Table 4:** Frequency of food consumption among participants (*n* = 330).

Food Item	I don′t eat	≤ 1 Time/Week	1–2 Times/Week	3–4 Times/Week	Daily	Mean score
Lean meat	51 (15.45%)	137 (41.52%)	98 (29.70%)	20 (6.06%)	24 (7.27%)	1.48
Fish	66 (20.00%)	196 (59.39%)	43 (13.03%)	14 (4.24%)	11 (3.33%)	1.12
Chicken	16 (4.85%)	32 (9.70%)	125 (37.88%)	75 (22.73%)	82 (24.85%)	2.53
Boiled egg	139 (42.12%)	80 (24.24%)	49 (14.85%)	31 (9.39%)	31 (9.39%)	1.20
Legumes	38 (11.52%)	93 (28.18%)	115 (34.85%)	40 (12.12%)	44 (13.33%)	1.88
Dairy products	92 (27.88%)	39 (11.82%)	65 (19.70%)	74 (22.42%)	60 (18.18%)	2.09
Nuts & seeds	63 (19.09%)	23 (6.97%)	72 (21.82%)	100 (30.30%)	72 (21.82%)	1.71
Vegetables	80 (24.24%)	75 (22.73%)	66 (20.00%)	41 (12.42%)	68 (20.61%)	1.82
Fruits	49 (14.85%)	53 (16.06%)	75 (22.73%)	59 (17.88%)	94 (28.48%)	2.29

*Note:* Values are presented as frequency and percentage for each consumption category. The mean score reflects average weekly consumption frequency per food item across participants.

### 4.5 Processed and Sugary Food Consumption

The results revealed that white rice and white bread were the most frequently consumed refined carbohydrate sources, with 46.36% and 36.06%, respectively, consuming them daily. In contrast, canned food had the lowest intake, as 56.36% of students reported never eating it, and only 4.85% consumed it daily. High intake of tea and caffeinated drinks with sugar was observed, with 37.88% consuming them daily, while sugar itself was rarely added directly, with 44.24% not using it at all. Fast food and sweets/cake were commonly consumed, with around 16% to 19% reporting daily consumption. Notably, energy drinks had a high non‐consumption rate of 46.97%, but 14.85% still consumed them daily. For more details, refer to Table 5.

**Table 5:** Frequency of consumption of processed and high‐sugar foods among participants (*n* = 330).

Food item	I don′t eat	≤ 1 Time/Week	1–2 Times/Week	3–4 Times/Week	Daily	Mean score
Fried egg	109 (33.03%)	82 (24.85%)	78 (23.64%)	21 (6.36%)	40 (12.12%)	1.40
White bread	60 (18.18%)	62 (18.79%)	54 (16.36%)	35 (10.61%)	119 (36.06%)	2.28
White rice	55 (16.67%)	24 (7.27%)	44 (13.33%)	54 (16.36%)	153 (46.36%)	2.68
Chocolate	80 (24.24%)	101 (30.61%)	71 (21.52%)	25 (7.58%)	53 (16.06%)	1.61
Fast food	38 (11.52%)	117 (35.45%)	92 (27.88%)	30 (9.09%)	53 (16.06%)	1.83
Sweets & cake	62 (18.79%)	92 (27.88%)	86 (26.06%)	26 (7.88%)	64 (19.39%)	1.81
Sugar	146 (44.24%)	79 (23.94%)	40 (12.12%)	14 (4.24%)	51 (15.45%)	1.23
Canned food	186 (56.36%)	88 (26.67%)	34 (10.30%)	6 (1.82%)	16 (4.85%)	0.72
Ketchup & mayonnaise	124 (37.58%)	116 (35.15%)	57 (17.27%)	12 (3.64%)	21 (6.36%)	1.06
Lard & animal skin	65 (19.70%)	135 (40.91%)	89 (26.97%)	25 (7.58%)	16 (4.85%)	1.37
Energy drinks	155 (46.97%)	68 (20.61%)	31 (9.39%)	27 (8.18%)	49 (14.85%)	1.23
Fizzy drinks	72 (21.82%)	112 (33.94%)	70 (21.21%)	37 (11.21%)	39 (11.82%)	1.57
Tea & caffeinated drinks with sugar	103 (31.21%)	44 (13.33%)	34 (10.30%)	24 (7.27%)	125 (37.88%)	2.07

*Note:* Values are presented as frequency and percentage for each consumption category. The mean score reflects average weekly consumption frequency per food item across participants.

### 4.6 Association Between Demographics and Diet Program Type

The results showed that monthly income and BMI category were significantly associated with the type of diet program followed (*p* = 0.01 for both), while gender had no significant effect (*p* = 0.61). Among those with lower income (<$385), 76.2% followed a normal eating routine, and only 2.4% consulted a nutritionist. Interestingly, students with higher BMI (obese) were more likely to follow non‐routine programs, as 14.3% of them received nutritionist‐guided plans, the highest rate across all BMI groups. In contrast, 84.2% of underweight participants adhered to a normal routine without professional guidance. These results suggest that income level and BMI status may influence students' dietary planning, with those at nutritional extremes showing more variance in approach. For extra details, refer to Table 6.

**Table 6:** Association between demographic characteristics and dietary pattern types among participants.

No.	Demographic variable	Category	Normal routine F (%)	Self‐designed program F (%)	Undernutritionist F (%)	Test result	*p* value
1.	Gender	Male	147 (71.0%)	54 (26.1%)	6 (2.9%)	*χ*² = 0.722	0.61
		Female	92 (74.8%)	27 (22.0%)	4 (3.3%)		
2.	Monthly income (USD)	< 385	32 (76.2%)	9 (21.4%)	1 (2.4%)	*χ*² = 6.814	0.01
		385–770	97 (70.3%)	33 (23.9%)	8 (5.8%)		
		> 770	110 (73.3%)	39 (26.0%)	1 (0.7%)		
3.	BMI category	Underweight (< 18.5)	16 (84.2%)	3 (15.8%)	0 (0.0%)	*χ*² = 8.763	0.01
		Normal (18.5–24.9)	151 (72.6%)	50 (24.0%)	7 (3.4%)		
		Overweight (25–29.9)	59 (72.8%)	21 (25.9%)	1 (1.2%)		
		Obese ( ≥ 30)	8 (57.1%)	4 (28.6%)	2 (14.3%)		

*Note:* BMI = body mass index. Dietary patterns include: Normal Routine Foods, Self‐Designed Program, and Under Nutritionist Supervision. Chi‐square tests are used to determine associations.

### 4.7 Differences in Food Consumption Patterns Across BMI Categories

The results showed that most food consumption patterns did not differ significantly across BMI groups; however, notable trends emerged in a few items. White bread consumption was significantly higher among underweight students compared to overweight and obese groups (*p* = 0.013), suggesting a greater reliance on refined carbohydrates among those with lower BMI. Additionally, a non‐significant trend indicated that obese participants consumed more chicken than others (*p* = 0.088), and underweight individuals tended to consume more fruits than overweight peers (*p* = 0.084). Effect sizes were uniformly small across all food items (η²p range: 0.003–0.033), indicating that while white bread consumption differed significantly across BMI groups (*p* = 0.013, *η*²*p* = 0.033), the practical magnitude of all observed differences was limited. No significant differences were observed in the intake of lean meat, dairy products, vegetables, or processed foods like fast food and sweets. For additional details, refer to Table 7.

**Table 7:** Comparison of food consumption patterns across bmi categories (*n* = 330).

No.	Food item	Underweight (*n* = 19)	Normal (*n* = 208)	Overweight (*n* = 81)	Obese (*n* = 14)	F‐value	*p* value	*η*²*p*	Post‐hoc results
A. Healthy foods									
1.	Lean meat	1.58 ± 0.84	1.38 ± 1.04	1.57 ± 1.11	1.93 ± 1.07	F(3,318) = 1.649	0.178	0.015	No significant differences
2.	Fish	1.37 ± 1.12	1.09 ± 0.81	1.10 ± 0.97	1.43 ± 1.28	F(3,318) = 1.160	0.325	0.011	No significant differences
3.	Chicken	2.74 ± 1.15	2.47 ± 1.08	2.53 ± 1.18	3.21 ± 0.98	F(3,318) = 2.197	0.088	0.020	Trend: Obese > Others^a^
4.	Boiled egg	1.42 ± 1.47	1.21 ± 1.31	1.22 ± 1.42	0.64 ± 0.75	F(3,318) = 1.010	0.389	0.009	No significant differences
5.	Dairy products	2.79 ± 1.32	2.06 ± 1.43	1.99 ± 1.63	2.00 ± 1.36	F(3,318) = 1.602	0.189	0.015	No significant differences
6.	Vegetables	2.05 ± 1.65	1.80 ± 1.43	1.72 ± 1.48	2.00 ± 1.41	F(3,318) = 0.368	0.776	0.003	No significant differences
7.	Fruits	2.79 ± 1.48	2.31 ± 1.35	1.98 ± 1.52	2.50 ± 1.40	F(3,318) = 2.234	0.084	0.021	Trend: Underweight > Overweight^a^
B. Processed/high‐sugar foods									
8.	Fried egg	1.89 ± 1.33	1.42 ± 1.34	1.28 ± 1.35	0.93 ± 1.14	F(3,318) = 1.671	0.173	0.016	No significant differences
9.	White bread	2.79 ± 1.51	2.43 ± 1.50	1.93 ± 1.60	1.64 ± 1.65	F(3,318) = 3.649	0.013	0.033	*Underweight > Overweight/Obese*
10.	White rice	2.68 ± 1.53	2.75 ± 1.50	2.72 ± 1.52	2.14 ± 1.75	F(3,318) = 0.705	0.550	0.007	No significant differences
11.	Chocolate	1.84 ± 1.46	1.63 ± 1.33	1.51 ± 1.43	1.29 ± 1.27	F(3,318) = 0.624	0.600	0.006	No significant differences
12.	Fast food	1.74 ± 1.28	1.86 ± 1.21	1.70 ± 1.25	2.43 ± 1.45	F(3,318) = 1.441	0.231	0.013	No significant differences
13.	Sweets & cake	2.42 ± 1.47	1.76 ± 1.29	1.74 ± 1.48	2.00 ± 1.52	F(3,318) = 1.518	0.210	0.014	No significant differences

*Note:* Values are presented as mean ± standard deviation. ANOVA was used to test for differences across BMI categories.

^a^ = marginal trend (*p* < 0.10), * = statistically significant (*p* < 0.05). Post‐hoc comparisons (Tukey) were only interpreted for significant or trending results. *η*²*p* = partial eta‐squared. Effect size interpretation: small (0.01), medium (0.06), large (0.14) per Cohen′s guidelines. All observed effect sizes were small, indicating limited practical differences across BMI categories.

### 4.8 Predictors of Following a Non‐Routine Dietary Pattern

Several lifestyle and behavioral factors were associated with increased odds of following a non‐routine dietary program. Students who always slept after midnight had 2.44 times higher odds of following a non‐routine diet compared to those who never did (OR = 2.44; 95% CI: 1.01–5.89; *p* = 0.047). Similarly, those who never achieved 7–8 h of sleep had twice the odds of following a non‐routine diet (OR = 2.07; 95% CI: 1.00–4.30; *p* = 0.050). Regular gym or swimming activity was associated with 49% lower odds of non‐routine dietary patterns (OR = 0.51; 95% CI: 0.31–0.85; *p* = 0.01). Additionally, always using electronic devices at bedtime was associated with doubled odds of following a non‐standard diet (OR = 2.03; 95% CI: 1.01–4.13; *p* = 0.048), and a family history of obesity was associated with 75% higher odds (OR = 1.75; 95% CI: 1.00–3.07; *p* = 0.049). For more details, refer to Table 8.

**Table 8:** Binary logistic regression analysis of predictors of following a non‐routine dietary pattern among participants (*n* = 330).

No.	Predictor variable	*B*	*SE*	Odds ratio (OR)	95% CI for OR	*p* value	Interpretation
1.	Gender (male vs. female)	0.41	0.24	1.51	[0.94, 2.42]	0.089	Males 51% more likely (trend)
2.	Age (per year increase)	−0.08	0.09	0.92	[0.77, 1.10]	0.372	No significant effect
3.	Income level						
	– Middle versus low	0.23	0.35	1.26	[0.63, 2.51]	0.511	No significant effect
	– High versus low	0.18	0.34	1.20	[0.61, 2.36]	0.596	No significant effect
4.	Sleep after 12 AM						
	– Sometimes versus never	0.52	0.47	1.68	[0.67, 4.22]	0.270	No significant effect
	– Always versus never	0.89	0.45	2.44	[1.01, 5.89]	0.047 *	Always staying up increases odds by 144%
5.	Sleep 7–8 h						
	– Sometimes versus always	0.34	0.28	1.40	[0.81, 2.42]	0.227	No significant effect
	– Never versus Always	0.73	0.38	2.07	[1.00, 4.30]	0.050 *	Poor sleep doubles odds
6.	Physical activity						
	– Walking versus none	−0.48	0.25	0.62	[0.38, 1.01]	0.055	Walking reduces odds by 38% (trend)
	– Gym/swimming versus none	−0.67	0.26	0.51	[0.31, 0.85]	0.01 *	Regular exercise reduces odds by 49%
7.	Electronic device use at bedtime						
	– Sometimes versus never	0.29	0.42	1.34	[0.59, 3.04]	0.490	No significant effect
	– Always versus never	0.71	0.39	2.03	[1.01, 4.13]	0.048 *	Always using devices doubles odds
8.	Family history of obesity	0.56	0.29	1.75	[1.00, 3.07]	0.049 *	Family history increases odds by 75%
9.	BMI category						
	– Normal versus underweight	−0.31	0.51	0.73	[0.27, 1.98]	0.543	No significant effect
	– Overweight versus underweight	−0.45	0.53	0.64	[0.23, 1.78]	0.395	No significant effect
	– Obese versus underweight	−0.28	0.67	0.76	[0.20, 2.84]	0.676	No significant effect

* *p* < 0.05 is considered statistically significant. B = unstandardized coefficient; CI = confidence interval; OR = odds ratio; SE = standard error.

## 5 Discussion

This study is the first to assess lifestyle behaviors and dietary patterns—including routine, preferences, and health‐linked predictors—among university students in Sulaimani, Iraq, with projections about behavioral implications. Our findings show that poor sleep hygiene, low physical activity, and unhealthy food consumption patterns are common among this group, particularly those with extreme BMI values. Students who slept after midnight and used electronic devices in bed were significantly more likely to follow irregular diets and avoid nutritionist guidance. Regular physical activity, especially gym or swimming, was linked to healthier routine adherence and better food choices. Notably, chicken was the most frequently consumed protein source, while fish, lean meat, and legumes were under‐consumed, indicating a skewed protein intake. Fruit intake was relatively better, but vegetable and dairy consumption lagged. Daily consumption of refined carbohydrates such as white rice and white bread was high and significantly greater among underweight students. According to our regression models, sleep patterns, BMI, and lifestyle behaviors significantly influenced whether students followed structured dietary plans or informal routines, which may shape future health risks.

The strategic objective of this study was to explore how sociodemographic, lifestyle, and behavioral factors relate to diet quality and health behaviors in emerging adults. University students represent a transitional demographic, increasingly responsible for their food choices yet often constrained by limited income, academic stress, and disrupted routines [33]. Among our sample, 45.45% had monthly incomes above $770, while 41.82% fell in the $385–770 bracket, underscoring economic variability. Our study also captured BMI variations, with 23.94% of students overweight and 5.76% obese. Nutritional guidance was followed by only 3.03%, showing limited professional dietary consultation. Academic pressures and lifestyle shift during university years have been linked to disrupted eating patterns and suboptimal health behaviors in similar contexts [10].

Therefore, the impact of student lifestyle on dietary quality and health risk extends beyond singular dietary choices and is deeply tied to behavioral routines. A substantial portion of students engaged in late‐night screen use, inadequate sleep, and low physical activity, behaviors shown to influence dietary decision‐making and metabolism [34]. For example, late‐night sleep was associated with a 2.44‐fold increase in non‐routine diet adherence, possibly due to disrupted circadian rhythms and impulsive eating patterns. Students using screens at bedtime had twice the odds of irregular diet behavior. Nutritional research confirms that irregular sleep and high screen time are linked to increased intake of sugary drinks and processed snacks [35]. Moreover, income level and family history of obesity significantly influenced whether students followed healthy routines, echoing findings from other low‐ and middle‐income countries [36]. Regular gym or swimming was associated with lower odds by 49%, supporting the role of structured physical activity as a protective factor [37]. These findings reinforce the interconnectedness of lifestyle routines and dietary planning among youth populations.

Previous studies examining college student nutrition have consistently shown that diet quality deteriorates during higher education, especially under financial strain [38]. One study found that more than 60% of university students in the MENA region consume fast food at least three times per week [39]. Our own data mirror this concern: 46.36% of participants consumed white rice daily, 36.06% consumed white bread daily, and nearly 20% consumed sweets and cakes every day. A large‐scale Iraqi youth survey linked daily fast‐food intake to a 40% higher risk of overweight status [3]. Research has also shown that skipping breakfast and irregular meal timing are associated with greater BMI and poorer academic performance [40]. Our study expands this evidence by revealing a nuanced link between sleep behavior, diet plan type, and body composition. Students who followed self‐designed or professional dietary plans were more likely to report better activity and sleep routines. Conversely, reliance on informal routines without guidance was dominant among underweight and obese individuals.

The results of our study—based on both group comparisons and logistic regression modeling—are consistent with prior research on health behavior clustering among university students. However, our estimates provide deeper insight by integrating predictors from sleep, physical activity, family history, and technology use. For instance, the strong relationship between bedtime screen exposure and non‐routine diet use highlights an emerging behavioral pathway that deserves further study. Additionally, our findings confirm that BMI extremes—underweight and obesity—are associated with more erratic eating behaviors and lower engagement with structured nutrition planning.

Following the observation of these interlinked behavioral patterns, it is concerning that only 3.03% of students were engaged in formal dietary programs. Without accessible, low‐cost, and student‐oriented interventions, unhealthy behaviors may persist and worsen over time. Student support systems currently focus more on academic advising than integrated wellness, leaving a significant service gap for young adults at risk of lifestyle‐related disorders. Immediate steps—such as sleep hygiene campaigns, subsidized nutrition counseling, and peer‐led activity groups—may help mitigate risk without requiring major structural changes. Preventive efforts during university years can yield long‐term benefits for individual health and national health systems.

Regional studies have documented rising rates of inactivity, poor nutrition, and stress among youth populations in Iraq and neighboring countries [41]. Inadequate campus infrastructure, high tuition‐related stress, and poor access to affordable healthy food contribute to the crisis. Recent global reviews confirm that students in the Middle East and North Africa are among the least physically active youth populations worldwide [42]. As many countries face rising costs and economic hardship, public universities must take on a greater role in facilitating health promotion among students.

In our study, the correlation between poor sleep, low activity, and irregular diets was strongest among students with extreme BMI values and those from low‐income families. These findings highlight the need for targeted interventions addressing health disparities even within relatively small, educated populations. Between 2020 and 2024, Iraq reported a 28% rise in university‐level overweight prevalence, mainly driven by dietary and activity changes post‐COVID [43]. Without action, it will be difficult for Iraq to meet national health objectives related to youth well‐being and non‐communicable disease prevention.

Lastly, several limitations should be considered when interpreting the findings of this study. The cross‐sectional design restricts causal inference, making it impossible to determine the directionality between lifestyle behaviors and dietary patterns. Self‐reported data may also be subject to recall bias and social desirability effects, particularly regarding sensitive variables like weight, sleep routines, or fast‐food consumption. Additionally, potential residual confounding from unmeasured variables such as parental education level, mental health status, and food environment access cannot be excluded. The use of convenience sampling and the overrepresentation of health‐related departments limit generalizability and introduce selection bias, as students in health disciplines may hold different attitudes toward dietary and lifestyle behaviors. Additionally, the sample was drawn from a single private university in Sulaimani, which may limit the generalizability of results to broader student populations across Iraq or other regions. Future research should consider longitudinal or intervention‐based designs to explore behavioral changes over time and test the effectiveness of targeted health promotion strategies.

## 6 Conclusion

This cross‐sectional study found that university students in Sulaimani exhibited suboptimal dietary patterns significantly associated with poor sleep hygiene, low physical activity, excessive screen time, and family history of obesity. Future longitudinal and intervention‐based studies are needed to establish causal relationships and evaluate the effectiveness of campus‐based health promotion programs in this population.

## Author Contributions

**Cheeman Salih Kakabra:** conceptualization, data curation, methodology, writing – original draft, Visualization, writing – review and editing. **Bushra Jabbar Hamarashid:** conceptualization, methodology, visualization, writing – review and editing. **Maisam Hama Murad Majeed:** conceptualization, methodology, visualization, writing – review and editing. **Ari Jamal Hassan:** conceptualization, methodology, visualization, writing – review and editing. **Sara Noori Mohammad:** conceptualization, methodology, visualization, writing – review and editing. **Bayan Omar Sharif:** conceptualization, methodology, visualization, writing – review and editing. **Prusha Soran Karim:** conceptualization, methodology, visualization, writing – review and editing. **Ara Othman Muhammed:** conceptualization, methodology, visualization, writing – review and editing. **Shan Hamza Muhammad:** conceptualization, methodology, visualization, writing – review and editing. **Savyar Ghafur Rashid:** conceptualization, methodology, visualization, writing – review and editing. **Bashir Mohammed Rasul:** conceptualization, methodology, Visualization, writing – review and editing. **Abdulmalik Fareeq Saber:** conceptualization, formal analysis, investigation, methodology, project administration, supervision, writing – review and editing. All authors have read and approved the final version of the manuscript. The corresponding author had full access to all of the data in this study and takes complete responsibility for the integrity of the data and the accuracy of the data analysis.

## Funding

The author(s) received no financial support for the research, authorship, and/or publication of this article. No external funding sources were involved in the study design; collection, analysis, and interpretation of data; writing of the report; or the decision to submit the report for publication.

## Disclosure

The lead author Abdulmalik Fareeq Saber affirms that this manuscript is an honest, accurate, and transparent account of the study being reported; that no important aspects of the study have been omitted; and that any discrepancies from the study as planned (and, if relevant, registered) have been explained.

## Ethics Statement

Ethical approval for the study was obtained from the Research Ethics Committee of the College of Nursing at UHD in January 2025.

## Consent

Written informed consent was obtained from all participants before they filled out the questionnaires.

## Conflicts of Interest

The authors declare no conflicts of interest.

## Supporting information

Supplementary Material.

## Data Availability

The data that support the findings of this study are available from the corresponding author upon reasonable request.
