# Supplementary material for: Assessment of Lifestyle and Dietary Patterns Among Students at the University of Human Development in Sulaimani City: A Cross‐Sectional Study 2025–2026
Source: Health Sci Rep. 2026 Mar 19;9(3):e72125. doi: 10.1002/hsr2.72125 (PMC13098071; doi:10.1002/hsr2.72125)
Supplement: Supplementary file 1 — Supplementary Material. [file HSR2-9-e72125-s001.docx]

**Assessment of Lifestyle and Dietary Patterns Among Students at the University of Human Development in Sulaimani City**

**Cross-Sectional Study Questionnaire — 2025–2026**

*All information collected is strictly confidential and used for research purposes only. Please tick (☐) the appropriate response.*

**Part 1. Socio-Demographic Data**

**1.** Age: __________ years

**2.** Gender:

☐ Male ☐ Female

**3.** Marital Status:

☐ Single ☐ Married ☐ Divorced

**4.** Monthly Family Income (USD):

☐ Less than $385 ☐ $385–770 ☐ More than $770

**5.** Academic Department:

☐ Accounting ☐ Business Administration ☐ Computer Science ☐ Information Technology (IT)

☐ English Language ☐ Law ☐ Medical Laboratory Science (MLS) ☐ Nursing

**6.** Academic Stage:

☐ 1st Year ☐ 2nd Year ☐ 3rd Year ☐ 4th Year

**7.** Place of Residence:

☐ Inside Sulaimani City ☐ Outside Sulaimani City

**Part 2. Body Mass Index (BMI) & Weight Management**

**1.** Body Weight: __________ kg

**2.** Height: __________ cm

**3.** BMI (calculated): __________ kg/m²

**4.** Do you use medication for weight gain?

☐ Yes ☐ No

**5.** Do you use medication for weight loss?

☐ Yes ☐ No

**6.** Have you undergone surgery for weight loss?

☐ Yes ☐ No

**Part 3. Past Medical History**

*Do you have a personal history of any of the following conditions?*

**1.** Type 2 Diabetes Mellitus:

☐ Yes ☐ No

**2.** Hypothyroidism:

☐ Yes ☐ No

**3.** Hyperthyroidism:

☐ Yes ☐ No

**4.** Polycystic Ovarian Syndrome (PCOS):

☐ Yes ☐ No

**5.** Asthma:

☐ Yes ☐ No

**6.** Helicobacter pylori (H. pylori) Infection:

☐ Yes ☐ No

**7.** Gastric Ulcer:

☐ Yes ☐ No

**8.** Celiac Disease:

☐ Yes ☐ No

**Part 4. Family Medical History**

*Does your immediate family (parents, siblings) have a history of any of the following?*

**1.** Type 2 Diabetes Mellitus:

☐ Yes ☐ No ☐ I don't know

**2.** Thyroid Disorder (Hypothyroidism or Hyperthyroidism):

☐ Yes ☐ No ☐ I don't know

**3.** Obesity:

☐ Yes ☐ No ☐ I don't know

**Part 5A. Dietary Pattern — General**

**1.** What type of diet program do you follow?

☐ Under Nutritionist Schedule ☐ Self-Designed Program ☐ Normal Eating Pattern

**2.** How many meals do you eat daily?

☐ One meal ☐ Two meals ☐ More than three meals

**Part 5B. Dietary Pattern — Food Frequency**

*Please indicate how often you consume each of the following food items:*

***A. Healthy Foods***

| **Food Item** | **I Don't Eat** | **≤1 Time/Week** | **1–2 Times/Week** | **3–4 Times/Week** | **Daily** |
| --- | --- | --- | --- | --- | --- |
| Lean Meat | ☐ | ☐ | ☐ | ☐ | ☐ |
| Fish | ☐ | ☐ | ☐ | ☐ | ☐ |
| Chicken | ☐ | ☐ | ☐ | ☐ | ☐ |
| Boiled Egg | ☐ | ☐ | ☐ | ☐ | ☐ |
| Legumes (beans, lentils, chickpeas) | ☐ | ☐ | ☐ | ☐ | ☐ |
| Dairy Products (milk, yogurt, cheese) | ☐ | ☐ | ☐ | ☐ | ☐ |
| Nuts & Seeds | ☐ | ☐ | ☐ | ☐ | ☐ |
| Vegetables | ☐ | ☐ | ☐ | ☐ | ☐ |
| Fruits | ☐ | ☐ | ☐ | ☐ | ☐ |

***B. Processed & High-Sugar Foods***

| **Food Item** | **I Don't Eat** | **≤1 Time/Week** | **1–2 Times/Week** | **3–4 Times/Week** | **Daily** |
| --- | --- | --- | --- | --- | --- |
| Fried Egg | ☐ | ☐ | ☐ | ☐ | ☐ |
| White Bread | ☐ | ☐ | ☐ | ☐ | ☐ |
| White Rice | ☐ | ☐ | ☐ | ☐ | ☐ |
| Chocolate | ☐ | ☐ | ☐ | ☐ | ☐ |
| Fast Food | ☐ | ☐ | ☐ | ☐ | ☐ |
| Sweets & Cake | ☐ | ☐ | ☐ | ☐ | ☐ |
| Sugar (added to drinks/food) | ☐ | ☐ | ☐ | ☐ | ☐ |
| Canned Food | ☐ | ☐ | ☐ | ☐ | ☐ |
| Ketchup & Mayonnaise | ☐ | ☐ | ☐ | ☐ | ☐ |
| Lard & Animal Skin | ☐ | ☐ | ☐ | ☐ | ☐ |
| Energy Drinks | ☐ | ☐ | ☐ | ☐ | ☐ |
| Fizzy Drinks (carbonated beverages) | ☐ | ☐ | ☐ | ☐ | ☐ |
| Tea & Caffeinated Drinks with Sugar | ☐ | ☐ | ☐ | ☐ | ☐ |

**Part 6. Sleep Patterns & Physical Activity**

***Sleep Patterns***

**1.** Do you sleep after 12:00 AM every night?

☐ Always ☐ Sometimes ☐ Never

**2.** Do you sleep 7–8 hours per day?

☐ Always ☐ Sometimes ☐ Never

**3.** Do you use electronic devices (phone, tablet, laptop) at bedtime?

☐ Always ☐ Sometimes ☐ Never

***Physical Activity***

**4.** Which regular physical activity (≥30 min, 5 days/week) do you practice?

☐ None ☐ Walking ☐ Gym or Swimming

*Thank you for your participation. Your responses are confidential and used solely for research purposes.*
